# Supplementary material for: Diversity in the Circulation of Influenza A(H3N2) Viruses in the Northern Hemisphere in the 2018–19 Season
Source: Vaccines (Basel). 2021 Apr 13;9(4):375. doi: 10.3390/vaccines9040375 (PMC8069444; doi:10.3390/vaccines9040375)
Supplement: Supplementary file 1 [file vaccines-09-00375-s001.zip › vaccines-1103289-supplementary.docx]

**Supplementary Table 1: GISAID sequences used in this manuscript**
